# Research on Financial Systemic Risk in ASEAN Region

**DOI:** 10.3390/e23091131

**Published:** 2021-08-31

**Authors:** Hong Fan, Renyun Liu

**Affiliations:** Glorious Sun School of Business and Management, Donghua University, Shanghai 200051, China; 2191056@mail.dhu.edu.cn

**Keywords:** systemic risk, node centricity, ASEAN region, minimum density method, interbank network

## Abstract

The research of financial systemic risk is an important issue, however the research on the financial systemic risk in ASEAN region lacks. This paper uses the minimum density method to calculate the interbank network of ASEAN countries and uses the node centrality to judge the systemically important banks of various countries. Then the DebtRank algorithm is constructed to calculate the systemic risk value based on the interbank network. By comparing the systemic risk values obtained through the initial impact on the system important banks and non-important banks, we find that the systemic risk tends to reach the peak in the case of the initial impact on the system important banks. Furthermore, it is found that countries with high intermediary centrality and closeness centrality have higher systemic risk. It suggests that the regulatory authorities should implement legal supervision, strict supervision, and comprehensive supervision for key risk areas and weak links.

## 1. Introduction

Based on history and development conditions, the financial development and structure of ASEAN countries have their own characteristics, and there are great differences among different countries. On the whole, the financial industry structure of ASEAN countries shows strong bank-dominated characteristics. The bank-dominated financial system is likely to lead to low resource allocation efficiency and increase bad debts of banks, thus increasing the systemic risk of the banking industry. Systemic risk refers to that the financial system is subjected to external shocks or changes in internal structure, which causes a common shock to the whole system. When the damage is very large, it will lead to financial crisis, which is an operating state between financial security and insecurity. At the same time, the banking-oriented financial system still lacks a perfect supervision mechanism. Therefore, under the background of the increasingly close relationship between the globalization and the financial institutions in ASEAN countries, it is urgent to study the important systemic banks and their systemic risks in ASEAN countries to help them discover the instability and potential systemic risk factors of the banking systems.

In the study of the systemic risk, Lehar [1] used the correlation of assets, asset volatility, and the capitalization value of bank management rights to construct the system risk index based on the number of default banks. Based on the balance sheet information, the IMF [2] measured systemic financial risks from capital adequacy ratio, leverage ratio, and liquidity. Illing and Liu [3] established the composite index of Canadian financial system, and Cardarelli [4] synthesized the financial pressure index of developed countries, and Balakrishnan [5] constructed the financial pressure index of emerging markets to describe the possibility of crisis in developing countries. Bartram [6] measured the systemic risk by calculating and comparing the changes in the return on stock prices of banks that have suffered losses during the financial crisis. The above literatures are based on the comprehensive index method to study the systemic risk. This method has high flexibility and operability. It does not make mandatory regulations on whether systemic risks will occur in the financial system. Instead, it starts from the internal causes of systemic risks, studies the internal links between various financial indicators and financial imbalances and market fragility, and then uses these indicators to build comprehensive indicators to measure the level of systemic risks. However, this kind of research also has some limits. The index selection depends on individual subject, and ignores the correlation between various financial institutions.

Brownlees [7] measured the contagion rate of the systemic risk according to the correlation coefficient of banks’ assets. Adrian [8] proposed CoVaR method to study the systemic risk, and Acharya [9] used SES index to measure the downside risk of a single financial institution in financial market turmoil. Karimalis [10] calculated ΔCoVaR based on the Copula function to test the factors leading to the financial systemic risk. Brownlees [11] constructed the SRISK index to measure the systemic risk spillover effect of financial market on a single financial institution. Billio [12] measured the risk spillovers between different financial institutions based on principal component analysis and the Granger causality test. Gravelle and Li used multivariate extremum theory to measure the systemic importance of a single or multiple financial institutions. Derbali and Hallara [13] used MES model to measure the systemic risk of European financial institutions. Sedunov [14] cited factors such as foreign equity exposure, securitization revenues, and bank size as contributing to banks' systemic risk exposure. For the ASEAN region, Rasoul looked at the cost structure and profitability of Singapore's banking sector. Alberto [15] explored the stability of the banking systems in Thailand, Malaysia, and Indonesia. Roengpitya [16] used the concept of CoVaR proposed by Adrian to measure the systemic risk of the Banking industry in Thailand. Vu [17] used VaR and DCoVaR methods to compare the loss level of Vietnamese enterprises under unstable events with the systemic risk of the whole market. Tansuchat [18] studied seven stock markets in six countries, Thailand, Malaysia, Indonesia, Vietnam, Philippines and Singapore, and their risk contribution to the ASEAN stock system by using the method of component expectation gap. Most of these literature studies are based on the systemic risk of a single country in the ASEAN region, and most of them have been conducted on countries with relatively developed financial markets, such as Singapore, Malaysia, Indonesia, Thailand, and the Philippines, while few studies have been conducted on Vietnam, Myanmar, Laos, Cambodia, and Brunei, which have slowed financial market development and relatively incomplete structure. If we consider the ASEAN region as a whole and analyze and compare the systemic risk status of all countries in the region, we will be able to better understand the region. At the same time, this part of the literature also has a point of commonality, that is, after the occurrence of a systemic event, from the perspective of the financial distress faced by a single financial institution, the impact of its loss on other institutions and even the entire financial system is analyzed and calculated. They take into account interinstitutional linkages, but do not intuitively reflect the structure of lending networks across the industry.

Using complex network methods to study the topological structure of financial market network provides a new research idea to solve the problem. Allen and Gale [19] studied the systemic risk based on the simple interbank network, and confirmed that the spread of contagion depends on the mode of interbank connection. They found that the complete network structure was more stable than the incomplete network, which provides a theoretical basis for the financial contagion network of the banking system. Gai [20] showed that the complexity and concentration of the interbank network structure increase the vulnerability of the banking system. Cont [21] measured the systemic importance of financial institutions using the “contagion index” and analyzes contagion risks in banking networks. In order to estimate the interbank network, Kanno [22], Fricke [23], Langfield [24] and many other scholars have used the maximum entropy method to estimate, but the network density estimated by the maximum entropy method must be too large. This is because the maximum entropy method assumes that banks in the network are fully connected to each other, whereas in reality two banks need to charge a large fee to lend to each other. The significance of the minimum density method proposed by Anand [25] for the first time in 2015 is to reduce the occurrence of this fee as much as possible to make the interbank network’s density smaller. This paper believes that the interbank lending matrix estimated by the minimum density method is closer to the real network structure, so this paper uses the minimum density method to estimate the interbank lending matrix of each country.

In most cases, the financial system only causes recession after external shocks, and will not cause a large area of institutional collapse. Inspired by the idea of eigenvalue center and PageRank feedback center, Battiston [26] proposed the DebtRank algorithm. He believed that when the buffer capital of financial institutions was affected, the recursive behavior of lending institutions would cause infectious losses to the whole banking system. The DebtRank algorithm solves the problem of contagion caused by the failure of institutions. It spreads in the interbank market by discounting the assets of institutions, and captures the contagion process of the banking system. Thurner [27] promoted the application of the DebtRank algorithm from the perspective of management, and pointed out that regulators can establish a reverse selection mechanism based on the DebtRank value. By calculating and regularly publishing the DebtRank value of each institution, it can help institutions to choose to borrow institutions with low DebtRank value when they need funds, which can effectively reduce systemic risks. Bardoscia [28] improved DebtRank algorithm with exponential relationship instead of linear relationship, and applied it to the study of European banks. Silva [29] used DebtRank and differential DebtRank algorithm to study the impact of periodicity on systemic financial risk.

Based on the above literature, the following problems need to be solved in the current research. (1) Most of the research focus on the systemic risk of the developed countries, while the research on the systemic risk of ASEAN countries are very scarce. (2) Most of the estimation methods of interbank network structure use the maximum entropy method, while the minimum density method will more truly estimate the structure of the real-world interbank network. (3) In terms of systemic risk measurement, most studies use the number of bank failures to measure the systemic risk. However, in the real world, bank failures are not common. Therefore, DebtRank method can better measure the systemic risk.

Therefore, in order to explore the lending network structure of banks in ASEAN countries, study the important institutions that affect the risk contagion among the networks, compare the systemic risks among banks in various countries, and analyze the relationship between systemic risks and important institutions, we have done the following work. We use the minimum density method to construct the interbank network of ASEAN countries. The network constructed by the minimum density method is more in line with the real characteristics. By analyzing the degree centrality, intermediary centrality, closeness centrality, eigenvector centrality, and assets ratio of each bank in the network, this paper studies the systemically important banks in the network. The systemically important banks refer to the banks that contribute greatly to systemic risk, and their stability determines whether the systemic risk occurs. At the same time, the DebtRank algorithm is constructed to analyze and calculate the systemic risk value of each country in ASEAN region, then whether there is a certain relationship between the systemic risk and node centrality is analyzed.

The contributions of the paper can be summarized as follows:

(1) At present, because there is little research on the systemic risk in ASEAN countries, this paper combines the minimum density method, the complex network method, and the DebtRank method to study the systemic risk of ten countries in ASEAN countries. (2) In this study, we can better understand the network structure, systemically important institutions, and systemic risks of banks in the ASEAN region. (3) According to the differences in the systemic importance of different institutions in different countries, this paper may put forward appropriate suggestions for different types of institutions to ensure the healthy development of the banking industry. 

## 2. Materials and Methods

### 2.1. Minimum Density Method

Suppose banking system X is closed and there are n banks in the banking system. The interbank network can be expressed as an N×N lending matrix, as shown in Equation (1):

(1) $$\begin{array}{l} X=\left[\begin{array}{ccccc} X_{11} & \cdots & X_{1j} & \cdots & X_{1N}\\ \vdots & \ddots & \vdots & \ddots & \vdots \\ X_{i1} & \cdots & X_{ij} & \cdots & X_{iN}\\ \vdots & \ddots & \vdots & \ddots & \vdots \\ X_{N1} & \cdots & X_{Nj} & \cdots & X_{NN} \end{array}\right]\begin{array}{c} A_{1}\\ \vdots \\ A_{i}\\ \vdots \\ A_{N} \end{array}\\ \;\begin{array}{ccccc} L_{1}\; & \cdots & \;L_{j}\; & \cdots & \;L_{N}\;, \end{array} \end{array}$$

(2) $$\begin{array}{l} \min_{X}\;c\sum_{i=1}^{N}\sum_{j=1}^{N}1_{\left[X_{ij}>0\right]}\\ \left\{\begin{array}{l} \sum_{j=1}^{N}X_{ij}=A_{i},\forall i=1,2,\cdots ,N,\\ \sum_{i=1}^{N}X_{ij}=L_{i},\forall j=1,2,\cdots ,N,\\ X_{ij}\geq 0,\forall i,j, \end{array}\right. \end{array}$$

where N denotes the total number of banks in the system, and $X_{ij}$ denotes the amount that bank i lends to bank j, and $A_{i}$ denotes the loan assets of bank i, and $L_{j}$ denotes the loan liabilities of bank j.

The matrix is solved by minimum density method. In the minimum density algorithm, parameter c is introduced, which represents the fixed cost of establishing connections between banks. That is, if there is an inter-bank connection, there will be a connection cost of c. Then the minimum density algorithm can be expressed as a constrained optimization problem for matrix X, as shown in Equation (2).

In Equation (2), the integer function 1 equals one only if the bank i lends to bank j. Because in the real banking system, borrowing between the two banks requires a lot of fees, so the significance of the objective equation is to minimize the cost of the occurrence, so that the banking network’s density is smaller. The constraint equation is to meet the constraints of the total amount of dismantled funds and the total amount of dismantled funds of each bank.

Then combining the information theory and economic incentive theory, two variables $AD_{i}$ and $LD_{i}$ are introduced:

(3) $$\begin{array}{l} AD_{i}=\left(A_{i}-\sum_{j}X_{ij}\right),\\ LD_{i}=\left(L_{i}-\sum_{j}X_{ij}\right), \end{array}$$

where $AD_{i}$ represents the surplus of the borrowed assets of bank i, that is, the surplus of the borrowed assets $A_{i}$ of bank i after borrowing out. $LD_{i}$ denotes the deficit of bank i' s borrowing debt $L_{j}$ after borrowing part. Introducing these two variables into the objective equation, the problem can be expressed as the maximization problem of Equation (4):

(4) $$V(X)=-c\sum_{i=1}^{N}\sum_{j=1}^{N}1_{\left[X_{ij}>0\right]}-\sum_{i=1}^{N}\left[\alpha_{i}AD_{i}^{2}+\delta_{i}LD_{i}^{2}\right],$$

where only by continuously reducing the values of $AD_{i}$ and $LD_{i}$ in sparse network X, can V(X) increases. $\alpha_{i}$ is a penalty term for deviations from the asset marginal and $\delta_{i}$ is a penalty term for deviations from the liability marginal. Numerically, the value of $\alpha_{i}$ is equal to the reciprocal of the square of the lending asset $A_{i}$, and the value of $\delta_{i}$ is equal to the reciprocal of the square of the lending liability $L_{i}$.

Links among banking networks are “mismatched”, which means small banks tended to choose to borrow with large banks rather than with small banks of the same size. To reflect the “mismatched” in real banking networks into our algorithm, we define a variable $Q_{ij}$, which represents the possibility of a connection between bank i and bank j:

(5) $$Q_{ij}=\max\left\{\frac{AD_{i}}{LD_{j}},\frac{LD_{j}}{AD_{i}}\right\}.$$

In Equation (5), the greater the “mismatched” between bank i and bank j is, the greater the possibility of connection $Q_{ij}$ is. In the process of algorithm iteration, the connection between bank i and bank j will be established first, so as to reflect the mismatch in the real bank lending network. 

Through Equation (5) to determine which banks in the iterative process of the algorithm to debit first, we need to calculate the specific amount of debit among the connected banks. At the same time, in order to ensure that the generated network X is both “sparse” and “mismatched”, a regulation mechanism is proposed. Suppose that we know the probability P(X) of all networks X distribution, and then we calculate the sum of two parts: The first part is the expected value of network: $\sum_{X}P(X)V(X)$. According to Equation (4), the larger the V value of the network, the less connections, which ensures the minimum density of the matrix. The second part is an entropy function: R(P| |Q) , which represents the closeness of network P and Q. According to Equation (5), Q matrix is the most “mismatched” matrix. Since the approximation degree of the two networks cannot be directly measured, we use the entropy function R to represent it. To ensure that network X is sparse and mismatched, this mechanism is represented as follows:

(6) $$\max_{P}\sum_{X}P(X)V(X)+\theta R(P|\;|Q),$$

where $R(P|\;|Q)=\sum_{X}P(X)\log P(X)/Q(X))$ and θ represents a negative coefficient. 

The above solution will get a closer to the real bank lending minimum density network.

### 2.2. Network Center Theory

Network center is the main promoter and controller of the evolution of financial network, which is the important node. Corresponding to the financial system, the network center is considered to be an important factor affecting the contagion and the characteristics. In this paper, we identify the network centers as systemically important institutions.

#### 2.2.1. Degree Centrality

Degree centrality is the most direct measure of node centrality in network analysis. The larger the node degree of a node is, the higher the degree centrality of the node is, and the more important the node is in the network. The node with the largest degree centrality is called the association center. The calculation formula is as follows:

(7) $$C_{D}(N_{i})=\sum_{j=1,j\neq i}^{N}x_{ij},x_{ij}=\left\{\begin{array}{l} 1,there\;is\;a\;connection\;between\;i\;and\;j,\\ 0,there\;is\;no\;connection\;between\;i\;and\;j. \end{array}\right.$$

Corresponding complex networks to the financial system, the degree center is the most widely connected banks, and the failure of the bank will have a broader impact.

#### 2.2.2. Betweenness Centrality

Betweenness centrality is an indicator that describes the importance of nodes by the number of the shortest paths passing through a node. It defines the ability of a network node to act as an intermediary for other nodes. The more the number of the shortest paths passes through a node, the more important the node is. The node with the largest betweenness centrality is called the infectious intermediary center. The calculation formula of intermediate centrality of nodes is as follows:

(8) $$C_{B}(N_{i})=\sum_{i\neq j\neq k}\frac{g_{jk}(N_{i})}{g_{jk}},$$

where $g_{jk}(N_{i})$ is the number of nodes i appearing on the shortest path between nodes j and k, $g_{jk}$ is the number of nodes j and k having the shortest path. In the financial network, the betweenness centrality of the nodes is a measure of the node contagion ability. The node with the highest betweenness centrality disappears, which can prevent more risk contagion paths. 

#### 2.2.3. Closeness Centrality

Closeness center is reflected in the degree of proximity between one node and other nodes in the network and the reciprocal of the average shortest path distance from one node to all other nodes represent the closeness centrality. That is, for a node, the closer it is to other nodes, the greater its closeness centrality is. Nodes with greater closeness centrality have better observation vision for the flow of information. Nodes with the largest closeness centrality are called close centers. The formula for calculating the closeness centrality of nodes is as follows:

(9) $$C_{C}(N_{i})=\frac{n-1}{\sum_{j=1,j\neq i}^{n}d_{ij}},$$

where n is the total number of nodes, and $d_{ij}$ denotes the shortest distance from node i to node j. According to the closeness centrality formula of nodes, it evaluates the importance of nodes according to the position of the nodes in the network. Corresponding to the complex network in the financial system, if a financial institution is closer to the center, the closer the connection between the financial institution and other financial institutions is, and when the institution is faced with the risk of failure by external shocks, it is more likely to quickly infect other financial institutions.

#### 2.2.4. Eigenvector Centrality

The meaning of feature vector center is that the importance of a node depends not only on the number of its neighbors (namely the degree of the node), but also on the importance of its neighbors. The eigenvector centrality is different from the degree centrality. The eigenvector centrality of nodes with many connections is not necessarily high because all the connections may have low feature vector centrality. Similarly, the high centrality of feature vector does not necessarily mean that it has high degree centrality. It has a few but very important connectors and can also have high eigenvector centrality. The node with the largest eigenvector centrality is called the influence center. Let $x_{i}$ be the center value of the eigenvector of node i, then the equation is described as follows:

(10) $$C_{E}(N_{i})=x_{i}=c\sum_{j=1}^{n}m_{ij}x_{j},$$

where n is the number of nodes, cis a proportional constant, and $x={\left[x_{1},x_{2},\cdots ,x_{n}\right]}^{T}$. When it reaches the steady state through multiple iterations, the following matrix form can be written:

(11) x=cMx

where M is the corresponding n×n adjacency matrix, and the meaning of adjacency matrix is, if two nodes are not directly connected, denoted as 0, otherwise denoted as 1. It is proved in the literature that if x is divided by the main eigenvalue λ of the adjacency matrix M, this equation can obtain a convergent nonzero solution, that is, $x=\frac{1}{\lambda }Mx$, so the constant $c=\frac{1}{\lambda }$, x is the eigenvector corresponding to the eigenvalue λ of the matrix M, and the element of the eigenvector is the eigenvector center value of each point in the graph. Feature vector centrality emphasizes the number and quality of neighbor nodes, and its score is the sum of its neighbor nodes. From the perspective of the financial system, the eigenvector centrality is suitable for describing the long-term influence of the financial institutions. For example, in the risk contagion, the $C_{E}(N_{i})$ of an institution is relatively large, indicating that the financial institution is more likely to be closer to the source of infection and needs to be prevented.

### 2.3. DebtRank Algorithm

DebtRank algorithm is constructed according to the complex network theory, inspired by the network feedback center and PageRank algorithm. It is a recursive contagion algorithm of institutional loss along the debt relationship. Its characteristic is to calculate the contagion of the interbank market without the collapse of the institution. DebtRank algorithm pays more attention to the correlation between banks. The value of network nodes (institutional assets) is also based on institutional relevance, which is consistent with the PageRank algorithm.

Assuming that the banking system is composed of n banks, the banks are represented by the nodes of the network, and the interbank lending is represented by the edges in the network. The loan from bank i to bank j is represented by $X_{ij}$ (there is a weight to the edge in the network), and the buffer capital of bank i is $E_{i}$ (the value of node i). Because the buffer capital is difficult to obtain, the owner’s equity of the bank is used to replace it. $w_{ij}=\min\left\{1,\frac{X_{ij}}{E_{i}}\right\}$ is defined as the impact factor of node j on i, that is, bank i lends to bank j, and bank j has an impact on bank i. If bank j fails, bank i loses $X_{ij}$, resulting in the reduction of the buffer capital of bank i or the collapse of bank i.

In order to prevent cyclic infection, we use the computational state to prevent cyclic calculation. Three states are defined for each node: normal state (U), recession state (D), and inactive state (I). The impact on the bank is defined as $h_{i}\in \left[0,1\right]$, $h_{i}$ is a custom constant. The larger the value, the greater the external impact on the bank. For the initial state (time step 0), the set of impacted banks is $s_{f}$, the state of banks in the set is D, and the state of banks not in the set is U. The impacted banks’ $h_{i}(0)=\psi$ and the un-impacted banks’ $h_{i}(0)=0$. At time step t, when the impact of bank i is transmitted along the network to the bank whose state is U at time step t−1, the bank state becomes D (calculating the influence of time step t+1). When infected to the bank with state D, the bank state becomes I (no longer calculated after one calculation). Other banks with state I remain unchanged. When calculating the impact at time step t, only banks with state U at time step t−1 participate in the calculation. This ensures that when the impact of bank i is infected, it only passes through each bank once.

**Figure 1:**
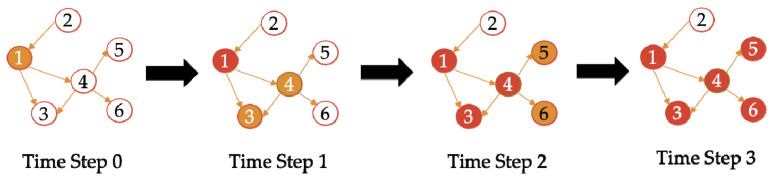
An example of DebtRank’s contagion process.

The contagion process is shown in Figure 1. At time step 0, node 1 is impacted, and the state of node 1 is D, while the state of other nodes is U. At time step 1, the impact of node 1 spreads along the network to node 3 and node 4 whose state is U at time step 0, and the states of node 3 and node 4 change to D. At the same time, the state of node 1 changes to I (it will not be calculated after one calculation). At time step 2, the impact of node 4 spreads along the network to node 5 and node 6, where their state is U at time step 1. The states of node 5 and node 6 change to D, while the states of node 3 and node 4 change to I. Node 1, whose state is I at time step 2, remains the same as I. At time step 3, the states of nodes 5 and 6 change to I.

The formula for calculating impact $h_{i}(t)$ is:

(12) $$h_{i}(t)=\min\left\{1,h_{i}(t-1)+\sum_{{j|s}_{j}(t-1)=D}w_{ij}h_{j}(t-1)\right\},$$

(13) $$s_{i}(t)=\left\{\begin{array}{ll} D, & h_{i}(t)>0,s_{i}(t-1)\neq I,\\ I, & s_{i}(t-1)=D,\\ s_{i}(t-1), & otherwise. \end{array}\right.$$

At the initial time step, the shock of $h_{i}(0)=\psi$ is carried out on a single bank, and $h_{i}(t)$ of each bank in the network is obtained by infection. Take the calculated values into the following formula to calculate the systemic risk value SR:

(14) $$SR(h(0))=\sum_{i=1}^{N}(h_{i}(t)-h_{i}(0))\varphi_{i},$$

where $\varphi_{i}$ represents the debt ratio of each bank, and SR(h(0)) indicates that the systemic risk is calculated under the initial shock of h(0).

### 2.4. Data

This paper studies the systemic risk of banks in ten countries of the ASEAN region at the end of 2018, and obtains banks’ data from the Bank Focus section of the BvD database. The types of banks are commercial banks, savings banks, cooperative banks, real estate and mortgage banks, investment banks, Islamic banks, central banks, multilateral government banks, and private banks. The banks are active and the ultimate owners of banks are national. Due to the availability of data, the following banks’ data are mainly obtained (see Appendix A for specific banks’ names and numbers). There are 3 banks in Brunei, 4 banks in Cambodia, 9 banks in Indonesia, 13 banks in Laos, 18 banks in Malaysia, 6 banks in Myanmar, 12 banks in the Philippines, 5 banks in Singapore, 12 banks in Thailand, and 11 banks in Vietnam. The total amount of assets and liabilities is obtained from the 2018 annual reports of these banks; the sum of bank loans, net advances, reverse repurchase, borrowed securities, and cash collateral are recorded as borrowing assets; the sum of bank deposits, repurchase agreements, borrowed securities, and cash collateral are recorded as borrowing liabilities.

After collecting the data, we find that the total lending assets and total borrowing liabilities of each country are not equal. In order to make the two equal, we assume another artificial bank, the (n+1) bank, to absorb the excess lending assets or borrowing liabilities. After the borrowing network is estimated, the removal of this artificial bank will not have any impact on the original banking network.

## 3. Results

### 3.1. Comparison of Network Topology Structure

**Figure 2:**
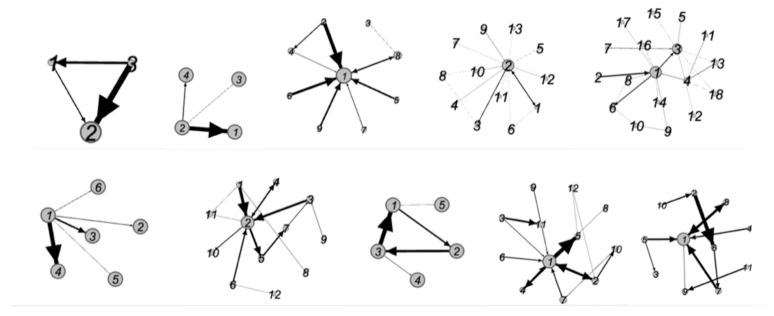
The network topology of the bank lending in ASEAN countries.

The minimum density method is used to construct the interbank lending matrix. Figure 2 shows the network topology of Brunei, Cambodia, Indonesia, Laos, Malaysia, Myanmar, the Philippines, Singapore, Thailand, and Vietnam. Among them, the topology of Brunei, Cambodia, Myanmar, and Singapore is relatively simple. The size of the banks in the graph is determined by the size of the entry, and the bank pointed by the arrow is the debt bank.

By comparative analysis of the network characteristics of various countries, we get the results shown in Table 1:

The density of the network is the number of connections in the network divided by all possible connections. Generally, large-scale networks are less dense than small-scale networks. As can be seen from Table 1, because of the small number of banks in Brunei, Cambodia, Myanmar, and Singapore, the network density of these four countries is larger than that of other countries, and the network density of the other six countries is less than 0.2. We believe that such networks are sparse, which conforms to the principle of minimum density method. Network diameter is the maximum distance between any two banks in the network, which is related to the stability of the whole network. It can be seen from Table 1 that the diameters of the 10 banking networks are all less than 5, and their network diameters are maintained at a low level, which also ensures the strong stability of the 10 networks. The average path length is the average path length between any two banks in the network (excluding itself to itself). It can be seen from Table 1 that the average path length of the 10 networks is in the range of [1,3], which reflects to a certain extent that the network has good contagion efficiency and good connectivity. 

### 3.2. Measurement of Systemically Important Banks

To facilitate a comparison of the importance of banks in the ten countries, we have divided the ten networks into two groups. Brunei, Cambodia, and Myanmar are in the first group, and the other seven countries are in the second group. The reasons for this group are as follows: in the three networks in the first group, there is a bank with betweenness centrality value of 1, that is, there is no direct connection between other banks except this bank. If the link is established, it must go through the bank with the largest intermediary centrality, that is, the infectious intermediary center mentioned in Section 2.2.2. In the second group, there is no bank with betweenness centrality value of 1, indicating that there are direct links between other banks. Table 2 and Table 3 respectively show the node centrality values and assets proportion of the important banks in the two groups of networks.

As can be seen from Table 2, Brunei, Cambodia, and Myanmar each have three network centers, namely, infectious intermediary centers, close centers, and influence centers, all of which are systemically important banks. In the Brunei, bank 1 is the infectious intermediary center and close center, and bank 2 is the influence center. In the Cambodian, bank 2 is the infectious intermediary center, and bank 1 is the close center and influence center. In the Myanmar, bank 1 is the infectious intermediary center and bank 4 is the close center and influence center. 

The three networks have the following commonalities: (1) The betweenness centrality of the infectious intermediary center is 1, indicating that there is no direct contact between other banks except the infectious intermediary center. If a link is established, it must go through the infectious intermediary center, which plays a pivotal role in risk spillover contagion. (2) There is a direct link between the influence centers and the infection intermediary centers, which meets the high-quality neighbor nodes, indicating that such centers are closer to the source of infection and need more prevention. (3) The high proportion of assets in systemically important institutions, especially infectious intermediary centers, suggests that the failure of such institutions can have a wider impact on the entire network.

Indonesia, Malaysia, the Philippines, and Brunei in Table 3 also have the three types of network hubs mentioned above. However, Laos, Singapore, and Thailand could not determine the influence center because their lending matrix did not have eigenvectors, so they could not calculate the eigenvector centrality of each bank in the network. In Indonesia, bank 1 is the infectious intermediary center and influence center, and bank 2 is the close center. In Laos, bank 2 is the infectious intermediary center and bank 1 is the close center. In Malaysia, bank 1 is the infectious intermediary center, bank 11 is the close center, and bank 3 and bank 6 are the influence centers. In Philippines, bank 2 is the infectious intermediary center, bank 8 is the close center, and bank 5 is the influence center. In Singapore, bank 3 is the infectious intermediary center, and bank 1 is the close center. In Thailand, bank 1 is the infectious intermediary center, and bank 11 is the close center. In Vietnam, bank 1 is the infectious intermediary center and influence center, and bank 10 is the close center.

These seven networks have the following commonalities: (1) A bank with high in-degree means that the bank is a debt bank of several banks, and is easy to affect other banks if the bank is unable to repay its debts due to default risk. A bank with high out-degree means that the bank is the creditor bank of several banks, and easily affected by the default cases of other banks; the bank with the largest sum of in-degree and out-degree is the association center. In Table 3, the in-degree is greater than the out-degree of all the association centers of the seven networks (the in-degree of Laos is equal to the out-degree), indicating that these association centers are more likely to act as debt banks and cause instability of the entire network once they are at risk. (2) The sum of the in-degree and out-degree of the infectious intermediary center is the maximum (the node centrality of non-important banks is not shown in the paper), which means that the association center and the infectious intermediary center are consistent. This is because the more edges a node establishes with other nodes, the greater the degree will be, and the more it can act as the path of the infectious risk. (3) The betweenness centrality of the infectious intermediary center is less than 1, which indicates that each node in the network has access to each other, so there is a high probability of risk contagion among banks; it also indicates that banks will share risks during risk contagion, which also makes the network relatively stable.

Based on the analysis of the above ten countries' systemically important banks, we can see that although the number of systemically important banks is different in different countries, each network has an association center with the highest degree, and each network has the infectious intermediary center, close center, and influence center. The association center and the infectious intermediary center are often consistent. The close center is not necessarily the node with the most links with other nodes in the network, but depends on the weight of the edges between the nodes. Influence center emphasizes more on the number and quality of neighbor nodes, and its score is the sum of the scores of its neighbor nodes. At the same time, systemically important banks tend to have a higher proportion of assets.

### 3.3. Comparison and Analysis of Systemic Risk

The initial impact on any bank i in any national banking network, $h_{i}(0)$ is 0.05, 0.1, 0.15, and 0.2 respectively; then according to the DebtRank algorithm, the bank lending matrix in Figure 1 is used as the data to calculate $h_{i}(t)$, and the values of $h_{i}(0)$are substituted into Equation (9) to obtain the systemic risk under the impact of bank i. The experiment is repeated n times (the total number of banks), and n systemic risk values are obtained in turn. The average value of n experiments is shown in Table 4. This average is defined as the systemic risk of the national banking network.

It can be seen from Table 4 that even when the minimum initial shock is 0.05, the systemic risk of Laos is higher than that of other countries, followed by Brunei. When the initial shock is less than 0.25, the systemic risk value is basically less than 5%, and we believe that events less than 5% are small probability events. Therefore, when the impact is less than 0.25, the probability of the systemic risk in Laos and Brunei is small, and the probability of the systemic risk in other countries is close to zero. However, when it receives shocks greater than 0.25, the probability of the systemic risk in Laos and Brunei is greater than 5%, which requires attention from relevant authorities in the country. As the risk value increases proportionally to the initial shock, it can be calculated that when the initial shock is 0.5, the probability of the systemic risk in Laos, Brunei, and Vietnam is greater than 5%; when the initial shock is 0.7, the probability of the systemic risk in Laos, Brunei, Vietnam, and Singapore is greater than 5%. The probability of the systemic risk in six other countries remains low.

Next, we explore systemic risk when hitting different banks.

**Figure 3:**
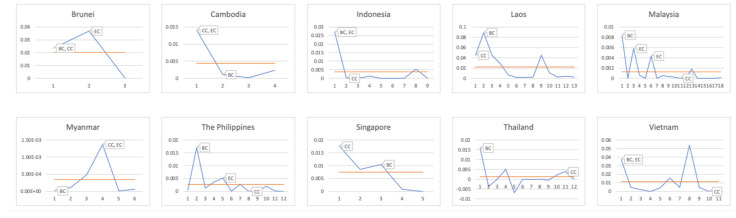
Systemic risk when hitting different banks.

As shown in Figure 3, the initial shock of $h_{i}(0)=0.1$ is carried out on each bank of each country in turn; the horizontal ordinate represents banks’ number, and the ordinate represents the systemic risk under the initial shock of 0.1 for this bank. The orange line represents the mean value of each point, which is the systemic risk value of this country as defined above. For the marked dots, BC is the infectious intermediary center, and CC is the close centers, and EC is the influence center. Therefore, the marked dots mean systemically important banks. 

It can be seen from Figure 3 that Brunei has the largest systemic risk when it impacts the influence center. Cambodia has the highest systemic risk when it impacts the close center and the influence center. Indonesia has the highest systemic risk when it impacts the infection intermediary center and the influence center. Laos has the largest systemic risk when it impacts the infection intermediary center. Malaysia has the largest systemic risk when it impacts the infection intermediary center. Myanmar has the largest systemic risk when it impacts the close center and the influence center. The Philippines has the largest systemic risk when it impacts the infection intermediary center. Singapore has the largest systemic risk when it hits the close center. Thailand has the largest systemic risk when it impacts the infection intermediary center. Vietnam has the highest systemic risk when it impacts the infection intermediary center and influence center.

As you can see, the systemic risk tends to peak when we have initial shocks to systemically important banks. 

Then we explore whether there is a certain relationship between the size of systemic risk and the size of betweenness centrality and closeness centrality. 

**Figure 4:**
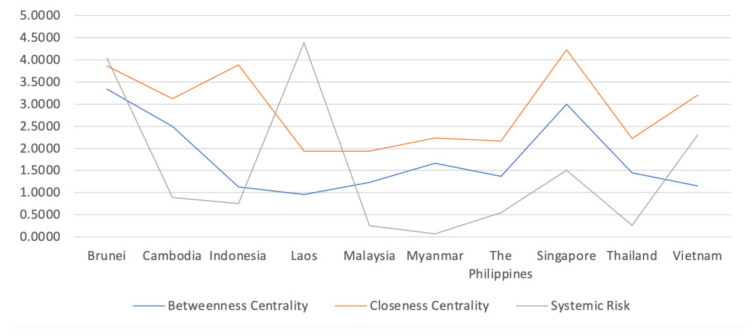
The trend of betweenness centrality, close centrality, and the systemic risk in ASEAN countries.

Figure 4 shows a trend map of the systemic risk, betweenness centrality, and closeness centrality in 10 countries, and the horizontal ordinate is 10 countries in turn, and the ordinate is the values of systemic risk, betweenness centrality, and closeness centrality. The betweenness centrality and closeness centrality are the mean of betweenness centrality and closeness centrality of banks in each network, and in order to facilitate comparison, the betweenness centrality and closeness centrality are expanded by ten times.

From Figure 4, we can see that Brunei, Singapore, Cambodia, and Myanmar are the top four countries in terms of intermediary centrality and near-centrality. As mentioned in 3.1, the network structure of these four countries is relatively simple and the network density is relatively large. In addition, Section 3.2 also mentions the existence of banks with intermediation centrality of 1 in Brunei, Cambodia, and Myanmar. Furthermore, Lenzu [30] shows that banks in more centralized networks are more likely to default and fail after a shock. As a result, we believe that more concentrated networks are more likely to create systemic risk. The trends of the three factors in the Figure 4 are also consistent with the conclusion of this study: the trend of systemic risk and the trend of betweenness centrality and closeness centrality are roughly similar. Countries with high betweenness centrality and closeness centrality have higher systemic risk.

## 4. Discussion

In this paper, the minimum density method was used to obtain the interbank network of ASEAN countries. Due to the small number of banks in Brunei, Cambodia, Myanmar, and Singapore, the network density of these four countries is larger than that of other countries, and the network density of the other six countries is less than 0.2. We believe that such networks are sparse. The diameters of the ten banking networks are all less than 5, and their network diameters are maintained at a low level, which also ensures the strong stability of the ten networks. The average path length of the ten networks is between the interval [1,3], which reflects to a certain extent that the networks have good connectivity. What is the impact of a well-connected network? Battiston [31] studied the impact of the financial accelerator mechanism and the interdependence mechanism in the financial network on the systemic risk, and believed that under the two mechanisms, the more complex the network structure is, the more likely the systemic risk will occur. Financial accelerators, where risk spreads over time, and interdependence, where risk spreads over space (networks), can both be considered transmission mechanisms. Namely, when a financial institution in t time is in financial trouble, the financial institutions of the creditor's rights agency in time (t+1) will make more restrictive conditions of the credit (financial accelerator), which make the agency's finances more vulnerable. This in turn leads to its neighbor banks absorb part of the impact to reduce the financial soundness of these neighbor banks (interdependence). In simple terms, when a bank has default risk, risk contagion is easy to occur among the well-connected networks, thus causing systemic risks. In addition, Nier [32] also believes that the impact of bank network connectivity is non-monotonous, that is, the initial small increase in connectivity will increase the contagion effect, but after a certain threshold, connectivity improves the shock absorption capacity of the banking system. When the risk spreads, each bank in the network will share the risk. The network has strong robustness.

Through the network, it was judged that there are systemically important banks in all countries. Although the number is different, there are association centers, infectious intermediary centers, close centers, and influence centers in the network. As the association centers and infectious intermediary centers, the capital flow with other financial institutions in the network is much higher than the transactions between other institutions. As Cajueiro [33] concludes, canters of capital concentration give other banks greater exposure, and thus have a greater probability of risk spillovers for such systemically important institutions. The association center and the infection intermediary center are often consistent, because the more edges a node establishes with other nodes, the greater the degree is, and the more it can act as the path of infection risk. The close center is not necessarily the node with the most links with other nodes in the network, but depends on the weight of the edges between the nodes. Influence center emphasizes more on the number and quality of neighbor nodes; its score is the sum of its neighbor nodes. At the same time, the proportion of assets of systemically important banks is generally high.

Then, the DebtRank algorithm was constructed to calculate the systemic risk value based on the interbank network, and the value is compared with 5% (small probability event) to determine that the banking systems of ASEAN countries are basically stable when the initial shock is less than 0.25. When the initial shock is greater than 0.25 and less than 0.5, the systemic risk probability of Laos and Brunei is greater than 5%; countries with systemic risks when initial shocks were greater than 0.5 or less than 0.7 increased, Vietnam; when the initial shock is greater than 0.7, countries with systemic risk increased, Singapore. The probability of the systemic risk in six other countries remains low. This is because the banking industry in Laos had a stable political and social environment for development only after the peaceful establishment of the country in 1975. However, it is still at a relatively low level of development: small credit scale and high non-performing loan ratio lead to the deterioration of bank assets; Single products and services, low efficiency of banking services are prominent problems, and it is difficult to meet the economic development and people's demand for financial services. The banking industry in Brunei and Vietnam has a similar problem: due to the short development history, the banking industry develops in a small scale, and the banking business mainly focuses on deposit, loan, and credit card business, which lacks diversification. Therefore, the operational risk of banks in these three countries is at a high level.

## 5. Conclusions

This paper used the minimum density method, complex network method, and the DebtRank algorithm to study the systemic risk in ASEAN countries, such as Brunei, Cambodia, Indonesia, Laos, Malaysia, Myanmar, the Philippines, Singapore, Thailand, and Vietnam et al. First, the minimum density method was used to construct the interbank network of ASEAN countries. By analyzing the interbank network, we found that the diameters of the ten banking networks are all less than 5, and their network diameters are maintained at a low level, which also ensures the strong stability of the ten networks. The average path length of the ten networks is in the range of [1,3], which reflects to a certain extent that the network has good contagion efficiency and good connectivity. Then the node centrality was used to judge the systemically important banks of various countries. Based on the analysis of the above ten countries' systemically important banks, we found that although the number of systemically important banks is different in different countries, each network has an association center with the highest degree, and each network has the infectious intermediary center, close center, and influence center. The association center and the infectious intermediary center are often consistent. At the same time, systemically important banks tend to have a higher proportion of assets. Finally, we analyzed the systemic risk in ASEAN countries. We found that the trend of systemic risk and the trend of betweenness centrality and closeness centrality are roughly similar. Countries with high betweenness centrality and closeness centrality have higher systemic risk.

According to the differences in the systemic importance of different institutions in different countries, the results of this paper may be used to optimize the differentiated regulatory governance of financial institutions in different countries. First of all, the key nodes in the banking network, namely the systemically important institutions analyzed in this paper, should be focused on supervision. Once they have default risk due to adverse shocks, the “domino effect” is likely to occur, which will bring crisis to the banking network and even the financial system. Therefore, for financial institutions with a large number of associations, short proximity, deep impact, and large proportion of assets, supervision should focus on and track their business conditions and risk management, and identify financial institutions with abnormal conditions in time to avoid causing systemic risks. Second, for non-critical nodes, these institutions should enhance their own operational robustness and risk resistance. In addition, a risk-warning mechanism can be established with the betweenness centrality, closeness centrality, and eigenvector centrality as the core. These three variables are related to systemic risk, because the more links an institution has with other institutions in the network, the shorter the path and the deeper the influence, the stronger the information transmission control ability of the institution, and the more systemic risks it can cause. Therefore, the above indicators can be used for early warning and management of systemic risks.

Furthermore, according to the countries' systemic risk differences, this paper may put forward some suggestions to promote the sound development of the banking industry. For countries with relatively high systemic risks such as Laos, Brunei, and Vietnam, the first step is to actively create an external environment for fair competition, which is conducive to providing richer financial products and more comprehensive financial services for the society and improving the overall development level of the banking industry. Moreover, to vigorously improve the level of information technology, to expand products and services to provide strong support in the front and back, and then expand the source of profit. Finally, only by strengthening the construction of risk control can the non-performing loan ratio be effectively reduced. After introducing mature risk analysis models from abroad and combining with their own needs to innovate, appropriate and effective risk analysis models can be formed, and then systematic risk warning and supervision can be conducted.

The systemic risk of a country defined in this paper is actually a numerical calculation of the shock value of the banks in the country, which can reflect the contagion effect of systemic risk caused by institutional correlation. At the same time, financial institutions are pro-cyclical. During the economic boom, they tend to expand credit, invest in riskier products, engage in more business to obtain higher profits, and increase inter-bank lending. It would also expose financial institutions to common exposures and improve their interconnectedness. Therefore, there is an endogenous causal relationship between the eruption of systemic risk and the excessive credit boom and the high correlation of financial institutions. As a result, the actual operating conditions of the banking sector should also be taken into account when measuring the systemic risk. In future studies, we will select multiple indicators that may cause systemic risks according to IMF [2] and other studies. These indicators will consider the pro-cyclicality and infectivity of systemic risks at the same time, and use principal component analysis to select important and unrelated indicators. After unifying dimensions, we will use entropy weight method to synthesize systemic risk composite index.

## Figures and Tables

**Table 1:** Comparison of the characteristics of the network in the ASEAN countries.

Country	Number of Banks	Interbank Connection	Network Density	Network Diameter	Average Path Length
Brunei	3	4	0.667	1	1.000
Cambodia	4	4	0.333	2	1.333
Indonesia	9	10	0.139	2	1.412
Laos	13	24	0.154	4	1.974
Malaysia	18	22	0.072	5	2.579
Myanmar	6	6	0.200	2	1.400
The Philippines	12	15	0.114	5	2.443
Singapore	5	5	0.250	3	1.750
Thailand	12	18	0.136	4	2.289
Vietnam	11	12	0.109	5	2.000

**Table 2:** Node centrality and asset proportion of the first group.

Country	Important Bank Number	In-Degree	Out-Degree	Betweenness Centrality	Closeness Centrality	Eigenvector Centrality	Assets Proportion
Brunei	1	1	2	1.0000	1.0000	0.1428	0.5329
	2	2	0	0.0000	0.0000	1.0000	0.2425
Cambodia	1	1	1	0.0000	1.0000	1.0000	0.6356
	2	1	3	1.0000	0.2506	0.1853	0.2758
Myanmar	1	1	5	1.0000	0.3404	0.0459	0.4888
	4	1	1	0.0000	1.0000	1.0000	0.0803

**Table 3:** Node centrality and asset proportion of the second group.

Country	Important Institution Number	In-Degree	Out-Degree	Betweenness Centrality	Closeness Centrality	Eigenvector Centrality	Asset Proportion
Indonesia	1	7	1	0.7500	0.0000	1.0000	0.1986
	2	0	2	0.0000	1.0000	0.0000	0.1542
Laos	1	1	2	0.0833	1.0000	—	0.4257
	2	11	11	0.9167	0.2217	—	0.2407
Malaysia	1	7	3	0.5176	0.0000	0.4064	0.3910
	3	7	1	0.3647	0.0000	0.9662	0.1178
	6	2	1	0.3059	1.44×10^−6^	1.0000	0.0469
	11	0	1	0.0000	1.0000	0.0000	0.0064
The Philippines	2	6	3	0.5926	0.0000	0.8073	0.2313
	5	1	1	0.2593	3.94×10^−6^	1.0000	0.0690
	8	0	1	0.0000	1.0000	0.0000	0.0245
Singapore	1	1	2	0.5000	1.0000	—	0.3879
	3	1	2	0.6667	0.6267	—	0.2734
Thailand	1	7	4	0.6944	0.0001	—	0.2559
	11	1	1	0.0000	1.0000	—	0.0057
Vietnam	1	5	1	0.4444	7.47×10^−7^	1.0000	0.1579
	10	0	1	0.0000	1.0000	0.0000	0.0436

**Table 4:** Systematic risk of ASEAN countries under different initial shocks.

Country	SR(0.05)/(%)	SR(0.1)/(%)	SR(0.15)/(%)	SR(0.2)/(%)	SR(0.25)/(%)
Laos	1.0966	2.1931	3.2897	4.3862	5.48275
Brunei	1.0083	2.0166	3.0249	4.0332	5.0415
Vietnam	0.5763	1.1526	1.7289	2.3052	2.8815
Singapore	0.3757	0.7515	1.1272	1.5030	1.87875
Cambodia	0.2212	0.4424	0.6637	0.8849	1.106
Indonesia	0.1873	0.3746	0.5619	0.7492	0.9365
The Philippines	0.1358	0.2716	0.4073	0.5431	0.679
Thailand	0.0666	0.1331	0.1997	0.2663	0.33275
Malaysia	0.062	0.1239	0.1859	0.2478	0.30975
Myanmar	0.0172	0.0344	0.0515	0.0687	0.086

## Data Availability

The data used to support the findings of this study are available from the corresponding author upon request.

## Appendix A

**Bank Number of Brunei**	**Bank Name of Brunei**
1	Bank Islam Brunei Darussalam Be
2	Autoriti Monetari Brunei Daruss
3	Baiduri Bank

**Bank Number of Cambodia**	**Bank Name of Cambodia**
1	ACLEDA Bank Plc
2	Cambodian Public Bank Limited
3	Union Commercial Bank Plc
4	Phillip Bank PLC

**Bank Number of Indonesia**	**Bank Name of Indonesia**
1	PT Bank CIMB Niaga Tbk
2	PT. Bank Panin, Tbk
3	Bank OCBC NISP Tbk
4	Bank Danamon Indonesia Tbk
5	Bank Permata Tbk
6	PT Bank BTPN Tbk
7	PT Bank Maybank Indonesia Tbk
8	PT BPD Jawa Barat dan Banten Tb
9	PT Bank Bukopin

**Bank Number of Laos**	**Bank Name of Laos**
1	Banque pour le Commerce Exterie
2	ICBC Limited Vientiane Branch
3	Lao-Viet Joint Venture Bank
4	Indochina Bank
5	Phongsavanh Bank Limited
6	ST Bank
7	BIC Bank Lao Co., Ltd
8	ACLEDA BANK LAO LTD
9	Saigon Hanoi Bank Lao Ltd
10	Lao China Bank Co Ltd
11	Banque Franco-Lao Ltd
12	ANZ Bank (LAO) Limited
13	Canadia Bank Lao Ltd

**Bank Number of Malaysia**	**Bank Name of Malaysia**
1	Malayan Banking Berhad—Mayban
2	Public Bank Berhad
3	RHB Bank Berhad
4	United Overseas Bank (Malaysia)
5	Bank Kerjasama Rakyat Malaysia
6	OCBC Bank (Malaysia) Berhad
7	HSBC Bank Malaysia Berhad
8	Alliance Bank Malaysia Berhad
9	Standard Chartered Bank Malaysi
10	Citibank Berhad
11	Bank of China (Malaysia) Berhad
12	Deutsche Bank (Malaysia) Bhd.
13	Kuwait Finance House (Malaysia)
14	Al Rajhi Banking & Investment C
15	Kenanga Investment Bank Berhad
16	Bangkok Bank Berhad
17	Bank Persatuan Malaysia Berhad
18	Bank of Nova Scotia Berhad

**Bank Number of Myanmar**	**Bank Name of Myanmar**
1	Central Bank of Myanmar
2	Kanbawza Bank Ltd-KBZ Bank
3	Ayeyarwady Bank Limited
4	Co-operative Bank Public Compan
5	Yoma Bank Limited
6	United Amara Bank Ltd

**Bank Number of Philippines**	**Bank Name of the Philippines**
1	BDO Unibank Inc
2	Metropolitan Bank & Trust Compa
3	Philippine National Bank
4	China Banking Corporation—Chi
5	Union Bank of the Philippines
6	Rizal Commercial Banking Corp.
7	Security Bank Corporation
8	Asia United Bank Corporation
9	Philippine Business Bank Inc
10	Philippine Bank of Communicatio
11	Philippine Veterans Bank
12	Producers Savings Bank Corporat

**Bank Number of Singapore**	**Bank Name of Singapore**
1	DBS Group Holdings Ltd
2	Oversea-Chinese Banking Corpora
3	United Overseas Bank Limited UO
4	BNS Asia Ltd
5	UOB Kay Hian Holdings Limited

**Bank Number of Thailand**	**Bank Name of the Thailand**
1	Bank of Thailand
2	Bangkok Bank Public Company Lim
3	Kasikornbank Public Company Lim
4	The Siam Commercial Bank Public
5	Bank of Ayudhya Public Company
6	TMB Bank Public Company Limited
7	United Overseas Bank (Thai) PCL
8	CIMB Thai Bank Public Company L
9	Kiatnakin Bank Public Company L
10	Industrial and Commercial Bank
11	Bangkok Commercial Asset Manage
12	KGI Securities (Thailand) Publi

**Bank Number of Vietnam**	**Bank Name of the Vietnam**
1	Sai Gon Joint Stock Commercial
2	Military Commercial Joint Stock
3	Saigon Thuong Tin Commercial Jo
4	Asia Commercial Joint-stock Ban
5	Vietnam Technological and Comme
6	Vietnam Prosperity Joint Stock
7	Saigon—Hanoi Commercial Joint
8	Ho Chi Minh City Development Jo
9	VietNam International Commercia
10	Southeast Asia Commercial Joint
11	Vietnam Export Import Commercia
